# ‘Like going into a chocolate shop, blindfolded’: What do people with primary progressive aphasia want from speech and language therapy?

**DOI:** 10.1111/1460-6984.12818

**Published:** 2022-11-30

**Authors:** Maria Loizidou, Emilie Brotherhood, Emma Harding, Sebastian Crutch, Jason D. Warren, Chris J.D. Hardy, Anna Volkmer

**Affiliations:** ^1^ Department of Neurodegenerative Disease Dementia Research Centre UCL Institute of Neurology University College London London UK; ^2^ Division of Psychology and Language Sciences University College London London UK

**Keywords:** Alzheimer's disease, communication partners, focus groups, primary progressive aphasia, semantic dementia, speech and language therapy

## Abstract

**Background:**

Primary progressive aphasia (PPA) describes a group of language‐led dementias. PPAs are complex, diverse and difficult to diagnose, and therefore conventional models of aphasia and dementia treatment do not meet their needs. The research evidence on intervention for PPA is developing, but to date there are only a few case studies exploring the experiences of people with PPA (PwPPA) themselves.

**Aims:**

To explore the experiences and opinions of PwPPA and their communication partners (CPs) to understand how speech and language therapy (SLT) services can better meet their needs.

**Methods & Procedures:**

A qualitative research approach was used whereby PwPPA and their friends or family members were recruited to participate in focus groups, via advertisements in the Rare Dementia Support PPA group newsletters. Consenting participants were allocated to attend one of four focus groups hosted on an online video conferencing platform. Participants were asked about their communication difficulties, and how SLT could address these needs. All meetings were transcribed, and data were examined using reflexive thematic analysis.

**Outcomes & Results:**

Six PwPPA and 14 CPs representing all three PPA variants and mixed PPA participated in the focus groups. Four main themes were identified during the analysis of the focus group discussions: (1) CPs’ burden, (2) adjusting to the diagnosis, (3) communication abilities and difficulties and (4) beyond language. A further 10 subthemes were identified.

**Conclusions & Implications:**

This study provides a greater understanding of the experiences and needs of PwPPA and their families in relation to SLT. This work underlines the importance of a person‐centred approach that considers the broader needs of both the PwPPA and the people around them. This will enable service providers to deliver SLT that meets the needs of PwPPA and their families and will also inform future research in this field.

**WHAT THIS PAPER ADDS:**

**What is already known on this subject**

- We know that PwPPA can maintain or even make improvements in word retrieval and speech fluency with SLT exercises. There is also developing evidence of the benefits of interventions such as CP training, communication aid support and other functional interventions.

**What this paper adds to existing knowledge**

- This study provides an understanding of the experiences and opinions of people living with PPA and their families in relation to SLT. Results demonstrate that PwPPA and their families have to navigate a complex journey, identifying strategies to support communication but also the influence of personality and other cognitive symptoms. SLT was useful, but not always available.

**What are the potential or actual clinical implications of this work?**

- This study will enable service providers to better plan, justify funding for and delivery of SLT that will meet the needs of PwPPA and their families. Most importantly this work underlines the importance of a person‐centred approach, incorporating the broader needs of the person with PPA and those around them.

## INTRODUCTION

Primary progressive aphasia (PPA) describes a group of language‐led dementias underpinned by frontotemporal lobar degeneration or Alzheimer's disease (Ruksenaite et al., 2021). People with PPA (PwPPA—this term will be used to refer to both people and a person with PPA) experience a progressive dissolution of language as the leading and dominant symptom impacting all aspects of daily living (Gorno‐Tempini et al., 2011; Marshall et al., 2018). It is estimated that there are several thousand people living with a diagnosis of PPA in the UK (Coyle‐Gilchrist et al., 2016). Though it constitutes only a small proportion of the total dementia burden, PPA is of disproportionate clinical importance because it tends to strike people in older midlife with a devastating impact on occupational and social functioning (Pozzebon et al., 2018; Ruggero et al., 2019).

Three major PPA variant syndromes are currently recognized in international diagnostic criteria (Gorno‐Tempini et al., 2011): non‐fluent/agrammatic PPA (nfvPPA), led by impaired speech articulation and/or sentence production; semantic variant PPA (svPPA), led by impaired word comprehension and loss of vocabulary; and logopenic variant PPA (lvPPA), led by word‐finding difficulty and reduced verbal working memory. Notably, a substantial proportion of PPA cases do not fit clearly into a single variant (‘mixed PPA’). As the disease progresses people can develop more global communication difficulties, alongside other cognitive and motor impairments (Marshall et al., 2018). Alterations in hearing due to impaired brain processing of sounds can compound communication problems in PPA (Hardy et al., 2017). Behavioural changes such as apathy can be a particularly distressing symptom for families to manage and affects overall engagement and motivation of PwPPA (Clarke et al., 2008). In fact, changes in social awareness and/or responsiveness can be more problematic for CPs than language symptoms and contribute to even greater levels of stress for both PwPPA and their families (O'Connor et al., 2021; Pozzebon et al., 2018). PPAs are complex, diverse and difficult to diagnose, and therefore conventional models of aphasia and dementia treatment do not meet their needs.

The impact of PPA has ripples beyond the person to affect their social and occupational networks. PwPPA report increasing social isolation and depression (Medina & Weintraub, 2007; Ruggero et al., 2019) and anxiety (Pozzebon et al., 2018). Spouses of PwPPA report feelings of gradual loss of a relationship and meaningful social interaction as well as the increasing dependency of their spouse with PPA on them for communication (Ruggero et al., 2019). Speech and language therapists report seeing PwPPA in their clinics who feel incompetent in conversations because of their language difficulties and family members who feel helpless to support them (Taylor et al., 2014).

In the absence of disease‐modifying treatments, non‐pharmacological approaches such as speech and language therapy (SLT) can help to maximize engagement and functionality in daily life (Kortte & Rogalski, 2013). The best evidence for speech and language interventions for PPA are for lexical retrieval therapies (Cadorio et al., 2017; Carthery‐Goulart et al., 2013; Croot et al., 2019; Jokel et al., 2014) and script training (Henry et al., 2016). When practised intensively, these impairment‐based interventions have been demonstrated to maintain and sometimes improve access and fluency in personally relevant words or phrases. More recently there has been a growing focus on functional communication interventions for PPA, which aim to support a person to execute an activity or participate in a life situation (Volkmer et al., 2020). Refining strategy use, such as gestures, or provision of communication aids, for example, have demonstrated the ability to maintain a person's ability to convey basic needs and preferences when accessing the community (Fried‐Oken, 2008). Communication partner (CP) training interventions teach both a PwPPA and their CPs strategies to maintain conversations, thus contributing to reducing isolation and facilitating the maintenance of relationships (Beeke et al., 2021). These functional intervention studies lack common outcomes, making it difficult to draw conclusions on the effectiveness of these interventions (Volkmer et al., 2020). This in turn inhibits the development of guidance for standard care in clinical practice and thus the translation of these interventions to the wider population of people with this diagnosis.

A survey of clinical practice in the UK indicated that speech and language therapists dedicate most sessions to functional communication approaches when working with PwPPA (Volkmer et al., 2019, 2020b). This survey also identified that there is much variability in the resources available at a local level, meaning the restrictive local criteria often precluded the offer of any SLT at all (Volkmer et al., 2019). This inequity in service provision highlights a lack of understanding of the needs of PwPPA.

Only a small number of case studies have focused on the subjective experiences of PwPPA and their families (Douglas, 2014, Pozzebon et al., 2017, 2018) identifying the progressive social isolation that both the person and their families experience on their disease journey. These case studies have also flagged the increasing dependence of the PwPPA on their loved ones in conversations (Pozzebon et al., 2017). One first‐hand account of living with PPA has emphasized the benefits of a wide range of personalized strategies that continually evolve as the disease progresses (Douglas, 2014). Other studies that have asked PwPPA and their families their opinions, have specifically focused on how speech and language therapists can support people to maintain relationships to inform the development of a CP training intervention (Volkmer et al., 2021).

Considering the views of PwPPA and their families can help inform design and delivery of clinical services as well as prioritize research agendas. Establishing the needs of people living with a diagnosis (service users) can be a valuable tool for managers and funders who are tasked with designing a service. This work can also assist clinicians, in the absence of a care pathway, in the adoption of evidence‐based interventions (in the broadest sense of the term ‘evidence‐based practice’ comprising three components: research‐based evidence, practice‐based evidence and informed patient preferences; Ruggero et al., 2020). This research aimed to address this issue by exploring the experiences and opinions of PwPPA and families to understand how SLT services can better meet their needs.

## METHODS

Qualitative research methods such as focus groups facilitate the exploration of experiences and opinions. The interaction between focus group members allows for constructive discussion and is ideal for stimulating new ideas and concepts (Steward et al., 2007) and in this case, exploring novel interventions. This study was conducted in compliance with the Consolidated Criteria for Reporting Qualitative Research Checklist (Tong et al., 2007). This project was part of the Rare Dementia Support (RDS) Impact Study which received approval from the UCL Research Ethics Committee (8545/004: Rare Dementia Support Impact Study).

### Participants

Convenience sampling was used to recruit participants from members of RDS. RDS is a UK‐based organization focused on empowering, guiding and informing people diagnosed with rare dementias, including PPA (https://www.raredementiasupport.org). Potential participants who had previously consented to being contacted about research studies were invited via email to participate in an online focus group on their own or with a friend or family member.

Inclusion criteria for PwPPA were: (1) adult (over 18 years of age); (2) self‐reported diagnosis of any form of PPA; and (3) able to participate in focus group discussions lasting up to 90 min, via videoconferencing. Inclusion criteria for CPs were: (1) live with and/or care for someone with PPA. Exclusion criteria included: a language impairment attributable to another pathology, such as stroke. People with varying degrees of ability and disease duration were included in the study. Due to COVID‐19 related restrictions it was not possible to meet face to face, thus meetings were held online via the remote conferencing platform GoToMeeting (LogMeIn Inc.).

Participants were invited to respond to a survey link, embedded in the invitation email, indicating their availability. They were consequently allocated to one of four focus groups (by authors CJDH, AV and ML), depending on: (1) their time availability; (2) PPA variant; and (3) being PwPPA or CPs. These divisions were made as the authors anticipated people with different PPA variants might benefit from different support strategies, for example, people with nfvPPA might need more time given to contribute due to their motor speech impairment, people with lvPPA might benefit from written prompts to support comprehension due to their reduced verbal working memory, and those with semantic impairment might benefit from further explanations and examples to support comprehension. Number of participants in each focus group ranged from three to eight, with zero to three PwPPA (Table 1). Focus group sizes were in line with literature recommendations of maximum six to eight participants, which allows for sufficient interaction and exchange of ideas, without discouraging quieter group members from participating, as may happen in larger groups (Amato, 2008). For reasons of pragmatism only four focus group meetings were planned (ML conducted this study as part of an MSc research project and was constrained by the timelines of her course).

**TABLE 1:** Participant allocation to focus groups

**Focus group number**	**Duration (min)**	**PPA variant**	***N*_PWPPA_**	***N*_CP_**	***N*_dyads_**	***N*_total_**
1	53.41	lvPPA	–	3	–	3
2	74.51	all	1	1	2	6
3	72.58	nfvPPA	2	4	1	8
4	68.18	svPPA	1	3	–	4

*Note*: PPA, primary progressive aphasia; lvPPA, logopenic PPA; nfvPPA, non‐fluent agrammatic PPA; svPPA, semantic variant PPA.

### Informed consent

Having completed the survey indicating their willingness to participate, participants were sent a participant information sheet (version V4 17‐12‐20) via email and invited to attend a remote consent session hosted on the GoToMeeting video conferencing platform. All consent sessions were video recorded, in line with the approved procedure outlined in the RDS Impact study protocol (Brotherhood et al., 2020).

### Procedures

All focus group meetings were also hosted on the GoToMeeting video conferencing platform. All participants attended the meeting from a quiet room in their homes . To increase participant engagement screen sharing was kept to a minimum. The focus groups lasted between 53 and 75 min (*M* = 67.17, SD = 8.27) and were facilitated by the first author (ML) a postgraduate student experienced in working with people with communication difficulties. An experienced co‐facilitator (AV) was present for the first two focus groups, to support the facilitator when needed. Participants were presented with the main research question: What speech and language therapy would be most useful to you? A structured topic guide was prepared in advance to ensure methodological consistency across focus groups. Planned prompts intended to encourage equal contribution of all participants and create space for PwPPA to share their opinions (see Appendix S1 in the additional supporting information). To ensure participants were able to access language related to the research question and focus group procedures, information was presented in an accessible format during the meeting. A slide presentation was prepared in advance (see Appendix SB online, for example), using accessible images designed for people with aphasia (Pearl, 2014). Stroke research on text formatting (spacing, font type and size) was used to guide the presentation of written information (Rose et al., 2011). All focus group meetings were video recorded.

### Data handling and management

Video‐recorded consent interviews, focus group discussions and participant personal information were stored securely in a Data Safe Haven on UCL servers, in compliance with General Data Protection Regulation (GDPR) legislation. During the transcription process, participants were consecutively assigned an identification number and any identifying information was anonymised. Data were stored on a secure, password‐protected database handled by RDS. Access to focus group data were restricted to members of the research team.

### Data analysis

The video‐recorded focus group meetings were transcribed automatically by the GoToMeeting platform. These transcriptions were thoroughly checked and amended by ML, through direct comparison of video recordings against transcriptions. Transcripts were documented in Microsoft Word and included spoken words, context, notable gestures and facial expressions. Reflexive thematic analysis was chosen to analyse data due to the nature of the research question: investigating participants’ experiences and opinions (Braun & Clarke, 2006, 2021). Data were analysed using an inductive approach, as outlined by Braun and Clarke (2020); data were collected specifically for this research question and coded without the intention to fit into pre‐existing themes. Six steps were applied to analysis (Braun & Clarke, 2006). In phase 1, the first author, ML, familiarized themselves with the data. There followed line‐by‐line systematic coding of interesting features, creating the initial codes (phase 2). In phase 3, data were organized into potential themes. In phase 4, co‐authors AV and CH were each given 50% of the uncoded transcripts to identify key semantic and latent codes. These were discussed and compared by the first author to assess for consistency and refine the themes. In phase 5, theme definitions and names were defined. Phase 6 included the final report writing and selection of extracts, as well as relating those to the research question and current literature. Data were analysed within a realist framework, in which a unidirectional relationship is assumed between participants’ experiences and language (Braun & Clarke, 2006). A realist epistemology focuses on individual psychology and assumes that language is a means to articulate meaning and experience (Braun & Clarke, 2006).

## RESULTS

A total of eight PwPPA and 16 CPs registered their interest to participate in the study. Two CPs did not wish to go through the consent interview process, and one PwPPA was unable to join the discussion at the allocated time and therefore did not participate. Seven PwPPA and 14 CPs therefore participated in the focus group, representing lvPPA (*n* = 4), nfvPPA (*n* = 7), svPPA (*n* = 5) and mixed PPA (*n* = 1). Participants presented with a range of 1–10 years since symptom onset (mean ± SD = 4.71 ± 2.54) and a range of 0–7 years since diagnosis (mean ± SD = 2.81 ± 1.70). A summary of cohort participant demographics are provided in Table 2 and individual participant characteristics in Table 3.

**TABLE 2:** Cohort participant demographics (total *n* = 21; this includes 13 individuals (three PwPPA and 10 CPs) and four couples (PwPPA with their CP))

**Participant characteristics**	**Number of participants (%)**
PwPPA	7 (33%)
CP	14 (66%)
*Relationship to PwPPA*	
Husband	5 (29%)
Wife	6 (43%)
Daughter	3 (21%)
*Gender*	
Male	9 (43%)
Female	12 (57%)
*Diagnosis (self‐report)*	
lvPPA	4 (24%)
nfvPPA	7 (41%)
svPPA	5 (29%)
mixed PPA	1 (6%)
*Years since symptom onset (self‐report)*	
< 3	4 (24%)
≥ 3 to < 6 years	8 (47%)
≥ 6 to < 10 years	4 (24%)
≥ 10 years	1 (6%)
*Years since diagnosis (self‐report)*	
< 3	7 (41%)
≥ 3 to < 6 years	8 (47%)
≥ 6 to < 10 years	1 (6%)
No report	1 (6%)^a^

*Notes*: CP, communication partner; PPA, primary progressive aphasia; lvPPA, logopenic variant PPA; nfvPPA, non‐fluent agrammatic variant PPA; PwPPA, people or person with PPA; svPPA, semantic variant PPA.

^a^Missing data are not calculated as part of the mean/SD.

**TABLE 3:** Individual participant characteristics

**Participant identifier**	**Focus group number**	**PPA variant**	**Years since diagnosis**	**SLT received**
CP01	1	lvPPA	1	9 months^b^
CP02	1	lvPPA	4	24 months
CP03	1	lvPPA	3	36 months^b^
CP04 and P02	2	nfvPPA	1	2 sessions
P01	2	svPPA	0	3 months
CP05	2	Mixed PPA	3	20 sessions
CP06 and P03	2	lvPPA	5	30 sessions
CP07	3	nfvPPA	2	6 sessions
CP08	3	nfvPPA	2	9 months
P04	3	nfvPPA	1	8 months
P05	3	nfvPPA	3	3 sessions^c^
CP10 and P06	3	nfvPPA	a	24 months
CP09 and CP11	3	nfvPPA	2	3 sessions
P07	4	svPPA	3	10 sessions
CP12	4	svPPA	4	a
CP13	4	svPPA	7	–
CP14	4	svPPA	4	6 sessions

*Notes*: CP, Communication partner; PPA, primary progressive aphasia; lvPPA, logopenic variant PPA; nfvPPA, non‐fluent agrammatic variant PPA; PwPPA, person with PPA; svPPA, semantic variant PPA.

^a^Missing participant information.

^b^Reported receiving SLT at irregular time intervals.

^c^Did not receive communication support.

### Themes

Four main themes were identified during analysis of the focus group discussions: (1) CPs’ journey, (2) adjusting to the diagnosis, (3) communication abilities and difficulties and (4) beyond language. A further nine subthemes were identified within these main themes. Table 4 presents an overview of the themes and subthemes.

**TABLE 4:** Overview of main themes and subthemes

**Themes**	**Subthemes**
1. CPs’ journey	1.1. *Responsibility of maintaining communication*
	1.2. *Shifting roles and responsibilities*
2. Adjusting to the diagnosis	2.1. *Negotiating healthcare support*
	2.2. *Coping strategies*
	2.3. *Need for education on PPA*
3. Communication abilities and difficulties	3.1. *Developing techniques to manage language symptoms*
	3.2. *CPs’ skills*
	3.3. *Experiences of SLT*
4. Beyond language	4.1. *Personally relevant SLT*

#### CPs’ journey

This theme describes the CPs’ journey from initially noticing the first symptoms. CPs were often the ones who first observed subtle changes in language and prompted their partner to seek medical advice:

The loss of nouns was the first thing that's happened and that was the first thing that prompted me to take her to the GP initially. CP02

In CPs’ experiences the progression of language symptoms were accompanied by other behaviours such as rigidity, repetitive behaviours and fixations. These behavioural symptoms were more difficult to manage than speech difficulties:

I think the speech would be […] reasonably manageable up to a point, but it's the […] the other behaviour aspect that I find the hardest. CP08

In managing these behaviours, CPs found consolation in separating their loved one from the disease. Thinking of PPA as a condition and separate from the person was helpful:

I think we have to say, it's the condition, not the person. CP01

Within this main theme two further subthemes were identified, namely: Responsibility of maintaining communication for CPs; and Shifting roles and responsibilities.

##### Responsibility of maintaining communication

In endeavouring to maintain communication CPs described various strategies they had discovered as supporting communication. Importantly they needed to refine these techniques over time, as their partners disease progressed, and the original strategy no longer worked:

As a partner, you work out what works and what doesn't, and you probably don't even realise that you're working it out. Then you just wait until it doesn't work anymore, and try something else. CP04

CPs’ responsibility for maintaining communication extended to preparing others on what to expect from a conversation with the PwPPA:

We went out with a couple, and I did brief them before we went down I said, you will find P02 blanks out because we are bantering […] and he can't keep up. CP04

Ensuring PwPPA felt confident in social settings was a priority for CPs who strove to prevent embarrassing situations. This extract captures the internal battle CPs faced in social settings when trying to encourage their loved one's independence but avoiding a sense of failure. As this CP discusses, the guilt is inevitable; she will either allow her partner to be put in an embarrassing situation or feel responsible for limiting his independence by doing things for him:

Just by default I always did it [order] because I felt embarrassed for both him and whoever was […] taking the order because neither of them could understand what the other one couldn't understand. I feel very torn at times. I don't want P02 to be put in a position where he's feeling uncomfortable and, embarrassed and whatever. So, I tend to try and snatch those moments away before they happen, but then I feel guilty, because I think perhaps it would have been okay. CP04

CPs also felt responsible for facilitating access to language practise at home and anticipating future needs. CPs described plans to establish technological supports, such as voice banking, in anticipation of future decline in communication skills. Yet CPs flagged this as an area they had limited knowledge of:

I've thought about it [voice banking], I wasn't sure how, or who was involved in it, and how you could actually become involved in that. CP07

##### Shifting roles and responsibilities

The diagnosis of PPA also caused shifts in relationship roles within each couple, leading CPs to assume new responsibilities, such as supporting PwPPA with their activities of daily living:

The kitchen was always her domain, and I just turned up and ate the food she prepared. So that role is reversed […], she's like a ghost behind me wanting to do stuff. So uh, you know I get her to peel the carrots. CP02

Despite assuming new responsibilities CPs felt that supporting the PwPPA to participate in tasks important. However, keeping people involved was not always easy and could lead to moments of frustration for the CP:

For God's sake, it's [the fridge] there. It's on your left. (Imitates husband) Huh? CP01

The variable and challenging symptoms, in combination with the numerous responsibilities CPs had to manage led them to appreciate patience. Loss of patience led to feelings of guilt and incompetence:

I just don't have the patience a carer needs and that makes me a very, very bad carer. CP03

#### Adjusting to the diagnosis

This main theme captured participants’ experience of how the PPA diagnosis changed their lives. At initial stages, loss, denial, frustration, and feelings of social isolation were common among most participants:

A complete loss of confidence because you knew you couldn't speak. And, social withdrawal […] if you withdraw and you shut down, then, you almost don't want to try anything. CP06

For some participants, coming to terms with a dementia diagnosis was more challenging than accepting the communication difficulties associated with the condition.

So […] I think, from our experience, it was really trying to find information about […] a word that we hated, which was you know, ‘dementia’ and the connotations that that had. CP05

As this participant explains, realizing the implications of the diagnosis signifies a big step towards acceptance. Only then can people move forward in finding ways to manage. However, participants who had been supported by speech and language therapists familiar with PPA and therefore skilled in educating people on the condition, adjusted to the diagnosis much more easily:

Our experience of SLTs is that they have learnt, through their various practices that they speak, in short sentences […] and use, appropriate words that people will understand. And I think that helped us get to grips with, with what PPA meant for *both* of us. CP06

This CP explains how a speech and language therapist helped them understand what PPA meant for both him and his partner and therefore acceptance of the diagnosis. Importantly, the use of simple language, rather than medical jargon was seen as an important facilitator in that.

Adjusting to the diagnosis for those without a partner was a different experience. The psychological burden of PPA appeared to be greater for this participant for whom living without a partner intensified feelings of exclusion. Witnessing the invaluable support of CPs heightened the anxiety of managing the later stages of the condition alone:

This is another experience for me […] I've got myself on my own, and I'm clearly fairly unique on that […] I'm just getting pretty worried about what the hell's gonna happen as things get worse. P07

Three subthemes were identified within this theme comprising Negotiating healthcare support; Coping strategies; and The need for education.

##### Negotiating healthcare support

In this subtheme, participants discussed the long journey of receiving a PPA diagnosis, which typically involved several years of being treated for other types of dementia or mental health issues. Many participants received false diagnoses and even pharmacological treatment for other conditions, such as anxiety. As illustrated by the following quotation, participants reported persevering for many years on their quests to the eventual diagnosis of PPA:

I've […] had problems since 2016 and I went to the GP countless times […] the consultant that I saw thought it was anxiety […] I've got a second opinion […] that resulted in me […] been [/being/] diagnosed in August 2020. P04

Delays in diagnosis also had financial implications, with people no longer eligible to receive aid for early onset dementias:

There was funding in (location) for younger onset dementias and she, by the time she was diagnosed, she, didn't qualify. CP12

Some participants were dissatisfied with the support from the healthcare system, particularly because it contributed to a time‐consuming diagnostic process that usually resulted in misdiagnoses and further hardships. When diagnosed correctly, participants had contrasting experiences with the quality of support services.

We've been very lucky to have [speech and language therapist's name] in both our lives really from the get‐go, so PPA sort of awareness has been forefront. CP05

As illustrated, participants who were treated by speech and language therapists familiar with PPA, reported a positive experience. However, receiving treatment from a professional familiar with the condition was attributed to luck, suggesting poor awareness amongst most healthcare professionals. Indeed, most participants explained that lack of SLT support stemmed from a lack of familiarity with PPA among speech and language therapists, due to the rarity of the diagnosis. Therefore, despite having access to SLT services, participants were not supported in a meaningful way:

she [speech and language therapist] said that, she felt that […] we probably knew more about semantic dementia [/svPPA/] than she did. CP12

The availability of services varied between geographic locations, with people in rural areas having no access to SLT at all:

Amazingly there is no SLT available in (town). It's all been, completely […] got rid of, decimated, (town) no longer have any facilities for it at all. CP07

This raised concerns about how the progressive nature of PPA shapes commissioners’ and professionals’ beliefs regarding the effectiveness of SLT. Participants discussed how the unavoidably declining outcomes in PPA possibly have implications for the funding of SLT services, and consequently the quantity and quality of SLT people receive.

If it's a degenerative disease, you're never going to have a positive outcome, are you? And I don't know how you convince somebody that, putting money into something that is [declining is worth it]. CP04

##### Coping strategies

In this subtheme participants discussed the value of coping strategies and explained how PwPPA continue to enjoy the things they love despite their difficulties, sometimes by adapting activities to their current abilities:

Although my husband can't read the newspaper properly, sometimes he's just having it there for a long session, to feel you've got that familiarity. CP08

CPs perceived engagement with familiar activities to be important to PwPPA, even if serving a different purpose. In the above example, despite reading difficulties that limited the person's ability to follow newspaper articles, the familiar activity of looking through the daily paper is still important. For some people with difficulties with reading, audiobooks were somewhat helpful:

I listen to audio books and, there's one thing that you can't do. Is ask them to repeat the last sentence. P05

This person describes the adjustment from reading books to listening to audio books, with the caveat that this had some limitations. The inability to have certain sentences repeated, in case the person requires clarifications or space to process the information, was an issue. This problem was shared by other participants who approached it in novel ways.

I read [him] books that are relevant to his life story […]. So I stop and start, if necessary. CP10

With the support of CPs, PwPPA were able to continue enjoy reading for longer, and had the option to stop, start or discuss sentences as necessary, ensuring they followed the thread of the story.

Watching television was an integral part of participants’ everyday lives and was important for them to continue with this activity despite language difficulties. Following complex television series and relating to numerous characters and storylines was particularly difficult for PwPPA. Simple shifts in watching habits, allowed people to still enjoy television. Game shows and particularly those that comprised repetitive routines or placed limited demands on language and working memory, made it easier for PwPPA to follow and enjoy:

Things like the ‘Tipping Point’. Those are the kinds of things that I do look at because there's only three sentences, […] I can then think right I can look at this then. P01

Most PwPPA preferred employing cognitive strengths in their leisure activities. Rather than focusing on their declining abilities, PwPPA chose to employ intact cognitive skills in their leisure time, which boosted their confidence:

Maths is always very, very high, which again makes me happy. P05

Likewise, involvement with a meaningful activity, like art or exercise helped PwPPA remain calm and keep motivated:

I do uh yoga every uh day uh six uh days a week […] I find that helpful. P04

##### Need for education on PPA

This subtheme captures the need for education around PPA symptoms and treatment options. Participants reported a need for information, to better understand their disease and thus, as this CP highlights, help guide them in managing the complex ‘puzzle’ of PPA:

Anything to guide along with it, and to make sense of a uh […] puzzle. Because not only are there the dementia aspects, then there's the, the aspect of speech, which is, uh, lacking in various ways with various people. CP01

For some participants information about PPA was felt to be more important than SLT interventions, particularly at the beginning of the disease journey:

The […] information criteria at the beginning […] was actually more important, probably than, how we communicate after 38 years of marriage […] It was really trying to find information about […] a word that we hated, which was you know, ‘dementia’ and the connotations that that had. CP05

Participants relied on professional guidance when it came to understanding their diagnosis and making treatment decisions, and this in turn depended on how this information and guidance was presented. Some professionals were felt to be more skilled in making information accessible and clear:

the neurologists I find, are not that easy—not that clear, they speak in long sentences, with long words. And, our experience of SLTs is that they have learnt, through their various practices that they speak, in short sentences […] and use, appropriate words that people will understand. And I think that helped us get to grips with, with what PPA meant for both of us. CP06

Participants also described a lack of knowledge of what SLT could offer. The metaphor used in the quotation really illustrates that participants feel SLT could have a positive impact, but they just did not know what or how:

it's rather like going into a chocolate shop, blindfolded. Uh, you, you don't know what there is to pick from? CP02

#### Communication abilities and difficulties

In this theme, participants shared their experience of communication skills after the diagnosis, commenting on the difficulties they now face. CPs observed that communicating needs was easier for PwPPA, compared with social conversations, in which emotional valence played a role:

Most of her language skills, now, aren't communication, they are needs, wants, information […] Pure conversation is a lot more difficult, but it does happen. Um and it can be with humour, um and often um with, fear or concern. CP05

Well‐rehearsed phrases or autobiographical memories in the form of stories, remained relatively unaffected even in advanced stages, when other language abilities had declined. This successful use of relatively simple phrases was perceived to boost the confidence of PwPPA in using their remaining language abilities:

She used to have various sentences and things that she would say on a regular basis. […] [these gave her some more confidence in terms of use of language […] if we were […] driving along the road, she would often say ‘this road's very busy, isn't it’ I mean she said it all the time. CP13

Importantly, participants agreed that individual conversations were easier to manage, compared with group settings.

The times when it was most difficult were the times, when we most wanted include her which is say, family events, when we'd all be talking […] at the same time. CP12

CPs voiced the need to include PwPPA in family events, although explained that such settings were particularly challenging. Abrupt topic changes and people talking simultaneously were judged as difficult for PwPPA to cope with, often causing frustration and eventually exclusion of people from conversation.

Three further subthemes were further identified within this theme, namely: Developing techniques to manage language difficulties; CPs’ skills; and Experiences of SLT.

##### Developing techniques to manage language difficulties

In this subtheme participants described the various approaches they had developed that helped them manage language symptoms. Different strategies were identified by individuals with different PPA variants, emphasizing the importance of strategies tailored to the underlying areas of language strength or difficulty. For example, limiting the number of terms used for a single semantic concept was an effective strategy for people with svPPA. This strategy resolved frustration and made PwPPA feel more capable:

Instead of referring to my dad as ‘dad’ or [name] or ‘your husband’, we just all called him [name] […] that, caused less frustration. CP12

People with nfvPPA commented on their difficulty with binary terms and misuse of words such as ‘yes’ and ‘no’. Use of gestures instead of words minimized confusion and frustration among participants, though even with such techniques patience was key.

I say all right, if you've heard me put your thumb up and if you've understood me, put the other one up. So I know you've both heard it and understood it. CP10

To further support understanding, CPs of people with lvPPA employed conversation prompts such as newspaper articles. This supported the PwPPA's ability to keep the topic in mind in the conversation with their CP:

[she'll say] ‘have you seen this’ ‘well yes, I have actually’ […] so we can have conversations like that, with a prompt in front of you like a newspaper article, or a picture. CP03

Other strategies were more universal, such as those that support telephone conversations, an area felt to be affected from the very early stages, for example. Combining audio‐visual support with video‐calling allowed others to see when the PwPPA had difficulties following the conversation, and conversely, allowed the PwPPA to rely on more than their auditory skills to follow the conversation:

We tend to facetime everybody now, so they see when he's […] struggling. CP04

Considering the different experience of PwPPA living with a CP from those living alone, there were differences in the types of strategies people described using. This PwPPA described often not speaking to anyone for long periods at a time and thus he developed strategies to help him practice speaking on his own:

I try and talk to myself when I'm doing it [cooking] about everything I pick up and do. P07

However, it was not only the progression of disease that impacted success of strategy use: environmental issues such as COVID restrictions were also felt to have an impact. Some felt the restrictions eliminated the pressure of having to act quickly. Though as restrictions were being lifted previously successful strategies were anticipated to require reviewing:

It's given time, to do things and we're, we're not in the big wide world where there's a lot of rushing and having to make […] snap decisions and talking quickly [… but] the strategies that worked this last year, may not work once we're out of this. CP04

##### CPs’ skills

In this subtheme participants elaborated on the value of CPs and the importance of being involved in SLT to refine their communication skills:

Certainly for us I got far more out of the therapy than (wife) did. CP03

Advice targeted directly at CPs was felt to be particularly helpful in developing their skills so they could, in turn, support the PwPPA to participate in conversation:

Therapy for me, and others to […] speak, and talk in way that made it easier for her to join in. CP13

Techniques applicable to CPs were perceived as more impactful to people's daily lives because CPs could more easily implement such strategies and train others in their social circle:

I need to be trained, not to be a SLT, but to train people in techniques and strategies that help P03. CP06

Spouses of PwPPA also highlighted how the CP's relationship with the person could benefit communication:

We've been married 48 years, we don't have to say anything sometimes. Just a look will do it! CP04

Drawing on a common experience, engaging the PwPPA in conversation facilitated a positive social experience. This CP was ‘briefed’ on the person's condition and therefore was advised on how to handle the situation, highlighting the importance of training and education:

He actually turned to P02 and said ‘right, do you remember […] the under 12s rugby tour?’ and actually fixated on something they had a common bond with. And they spoke about that between themselves for a very long time, very lucidly. But that was someone who had been briefed to know what to do. CP04

Patience was identified as a crucial quality in both CPs and PwPPA in resolving communication breakdown:

If you have the confidence and you know that person well to ask them to repeat it, and if they've got the patience to deal with that. CP08

Interestingly, while most CPs assumed responsibility for adjusting their communication style to the abilities of PwPPA, in this example, the relationship between the person and the CP is mutual.

##### Experiences of SLT

In this subtheme participants shared their experience of the range of SLT, that helped or did not help them. Group sessions were reported as beneficial, allowing people to collect a number of ideas, though it was noted that given the variability within PPA symptoms this also meant it was sometimes difficult to relate to others:

The trouble with the group activities is that initially, I found they were useful to me, picking up tips from different people, but one of the great problems with this disease is, it's pretty difficult to find any two people that have the same, exactly same problem. CP03

PwPPA sought to strengthen their language skills through practicing word training. However, as illustrated by this quotation this was not necessarily enjoyable. The evident perseverance of people with svPPA with word training, despite their discontent, would suggest the technique is beneficial:

I have been doing quite a lot of […] word training. […] I do about an hour every morning which is […] kind of painful. P07

Use of scripts was common practice, though participants reported variable success. Participants explained that this method imitates natural conversation, but even slight deviations from the script caused confusion, as illustrated by the following example:

It might work while you're writing it down, but by the time you need to do it it's, you know, it's gone or […] if somebody answers with not the answer you're expecting […] that throws you off completely. CP04

Participants also commented on how restrictions imposed over the past year affected the delivery of SLT. Participants raised concerns around engagement with online therapy, which was less engaging for PwPPA, therefore limiting its effectiveness:

The problem with remote stuff like this, is that it's very easy to get distracted by looking out the window or something. CP03

However, in some cases COVID‐19 resulted in an easing of previous limitations:

They would only see certain people within their locality, [but] because of lockdown they had opened up to (video conferencing). CP09

Beside language support, speech and language therapists helped with a range of PPA symptoms, including behavioural symptoms. Behavioural symptoms were challenging for CPs to manage, but as this participant explains, simple techniques taught in SLT supported her in managing fixations and minimizing frustration, allowing the conversation to progress.

One thing [the speech and language therapist] taught me […] when this fixation comes, she said, uh ‘we will park that idea to the side’ and that it's so lovely because it's actually helped. CP01

#### Beyond language

In this theme participants emphasized how each person is unique in their needs, preferences and symptoms, elaborating on the difficulties they face beyond communication. The individuality of each person was perceived to create unique needs which should be targeted in SLT:

I think the individuality of each person is, is important because there are different needs with each person. CP01

Ageing‐related decline in hearing and cognitive functions, in both PwPPA and CPs was believed to exacerbate miscommunication and frustration:

My husband's hearing isn't […] wonderful and, and perhaps the assumption that what I'm going to just say, is something else […] He does often misinterpret what I've said. CP08

Fatigue affected people's clarity and determined how they structured their day, with important or cognitively demanding tasks being done in the morning.

I'm pretty certain my brain gradually gets worse through the day. Um, so it's best to do it [word training] in the morning. P07

Even with simplification of language, PwPPA had difficulties with decision‐making, possibly suggesting an underlying difficulty beyond language comprehension.

I can say ‘do you fancy the beef?’ ‘yes’, ‘do you fancy the fish?’ ‘yes’, ‘do you fancy the chicken?’ ‘yes’ (laughs). ‘Uh okay which would you prefer?’ […] uh and you know nothing! CP02

One further subtheme was identified within this main theme, namely: Personally relevant SLT.

##### Personally relevant SLT

Participants valued professional guidance in navigating the numerous SLT approaches yet felt that the choice of which one to pursue should be theirs and should be selected with respect to their individual strengths, difficulties and needs. Overall, a personally relevant approach to SLT was important to all participants:

I think what you'd have to have is like a massive spider diagram, sort of like this is where we [are] and these are the 10 answers that somebody could give from here and then spread out. CP04

As evidenced by this quotation, timely intervention was key, with participants having little expectations from SLT at advanced disease stages.

A couple of years ago or maybe, or a year ago, it might have helped, but […] my husband […] can't follow anything right now […] So, listening to someone just doesn't happen. CP14

As this CP explains, reinforcing weaker language domains early in the disease course can be more valuable than introducing novel techniques when communication abilities are already struggling. Therefore, considering and utilizing intact abilities in SLT seems to be important:

Perhaps it's [writing] something that I would gradually, introduce in our lives now. So […] when it […] does–is needed, it's a natural thing to do. […] But if we've never done it, then to introduce it at a time when, everything else is, struggling, then, perhaps that's not a great time to be introducing that. CP04

When it came to reading exercises, some CPs shared concerns that their loved ones were possibly dyslexic before their diagnosis, explaining why reading was never something they enjoyed.

Now trouble is [name] is likely dyslexic as well, so he's never liked reading. So when they [scripts] were written down, he found it a bit hard. CP01

PwPPA discussed facing various difficulties with writing, agreeing that SLT aimed at improving writing skills would have limited benefit. Difficulties varied from physical difficulties with holding a pen, deterioration of handwriting or difficulty in placing letters in the correct order, suggesting various possible mechanisms.

My handwriting is is, is so tiny […] It used to be large and now it's tiny […] I can't read my own writing sometimes. P06

However, in others, writing key words, supported PwPPA in keeping a concept in mind or communicating a message they struggled to express verbally. For others, writing down important information like the names of their children supported their memory of important words.

She's not able to write coherent sentences and there will only be a few words in there, but those words mean something […] like the names of our kids. CP13

While some PwPPA were disinterested in the use of technology, others were dissatisfied with the lack of technological innovation in SLT. Tailoring SLT interventions and degree of technological reliance to the person's preferences related to motivation and adherence to treatment:

They [speech and language therapists] made me carry around this big box of, paper bits. Which, yeah, in the old days that, that was the way to do it. But now I think […] an app, works really well in some ways. P07

Participants emphasized the value of SLT around activities PwPPA are still involved in or used to enjoy, to keep them interested and motivated:

Some sort of therapy around the specific activities that the person, is still engaged in […] some, sort of conversation that latches onto something that the, the person is still doing actively. CP13

**FIGURE 1:**
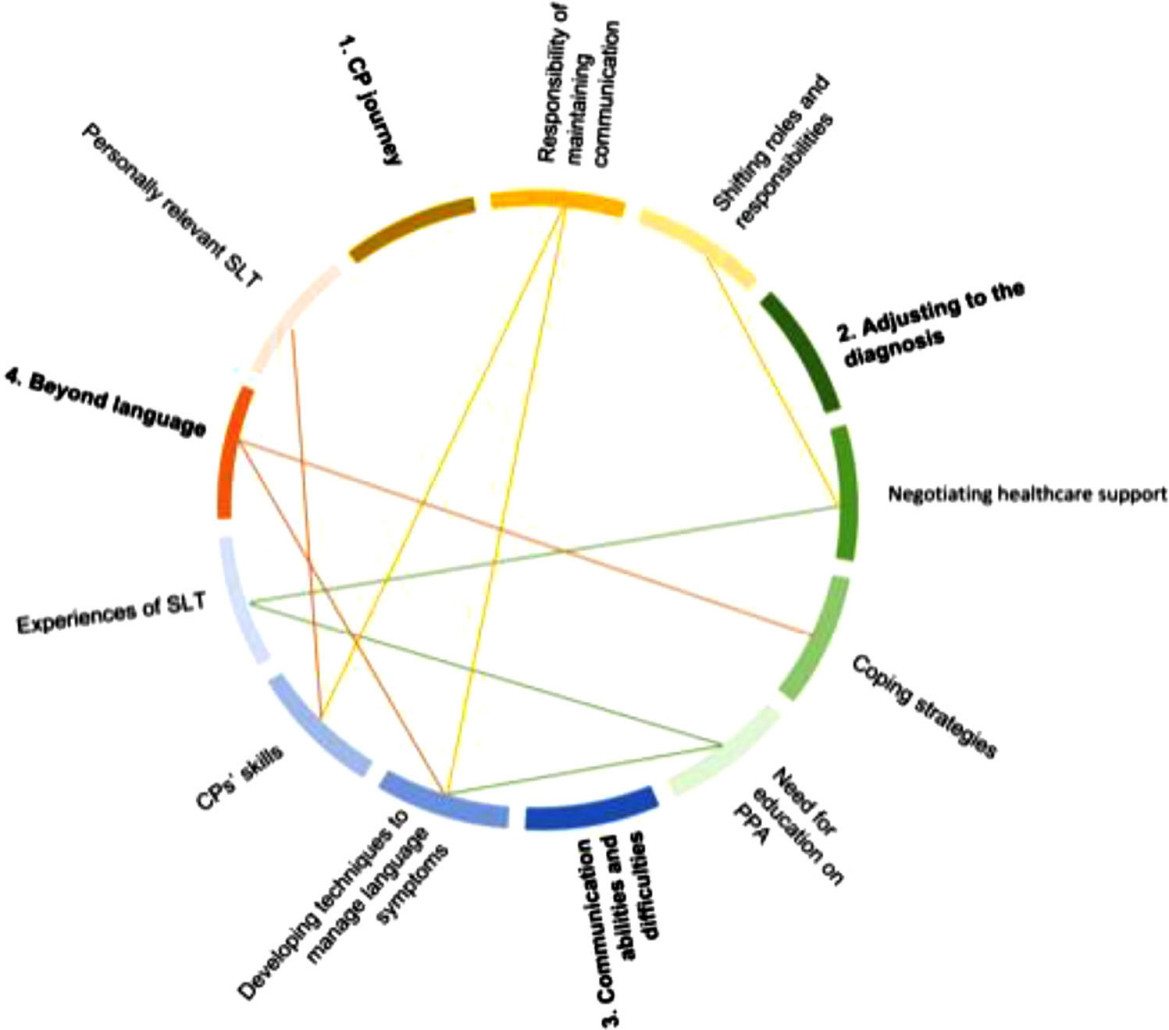
Schematic representation of the relationships between themes and subthemes. The main four themes are numbered, and yellow, green, blue and red represent the overarching themes and respective subthemes. Coloured lines reflect relationships between certain themes or subthemes as illustrated by participants during discussion. [Colour figure can be viewed at wileyonlinelibrary.com]

The relationships between themes and subthemes, as illustrated in Figure 1, is critical to understanding the individuals experience and consequently delivering a personally relevant approach. In Figure 1 the metaphor of coloured threads helps the reader tie together the relationships and interactions between themes. Each colour represents a main theme, whilst the differing shades represent the subthemes. The threads have been connected to demonstrate the relationships identified in these focus groups.

## DISCUSSION

The aim of this study was to understand the experiences and opinions of PwPPA, and their friends and family members, and how SLT services have met and could have better met their needs. Results demonstrated that PwPPA and their families must find ways to adjust throughout their journey, and though they find the support of SLT helpful, it is not always available. People shared different experiences and different strategies, often describing additional factors such as personality or other cognitive symptoms as influencing these. Finally, the important role and needs of CPs in supporting communication must not be overlooked in SLT.

Individual participants highlighted different communication strategies that were more useful to them depending on their diagnosis, disease journey and personal preferences (Table 3). This need for a personalized and person‐centred approach to management of communication difficulties is a central principle in a recent international consensus study on SLT for PwPPA (Volkmer et al., 2022). Whilst some of the strategies listed in Table 5. have been described in the research literature for PwPPA, such as using physical prompts (Mooney et al., 2018) and gestures (Fried‐Oken, 2008), others are more a reflection of what participants with PPA have developed independently and may have refined with feedback. This reinforces findings from a recent review of functional interventions for PPA, which identified that refining strategies people have already developed is a common foundation for this type of therapy (Volkmer et al., 2019).

**TABLE 5:** Communication strategies employed by participants

**Description**	**Diagnosis**
Use of physical prompts (e.g., newspaper articles)	lvPPA
Use of dichotomous questions (yes/no)	lvPPA
Writing down key words to facilitate retention of the conversation topic	lvPPA, nfvPPA
Video calls instead of telephone	nfvPPA
Repetition of a verbal message (by PwPPA or CPs)	nfvPPA, mixed PPA
Thumbs up to show agreement	nfvPPA, mixed PPA
Limiting number of terms used for a semantic concept	svPPA
Numbers to replace semantic concepts	svPPA
Describing an activity while doing it	svPPA

Early introduction of impairment‐based maintenance interventions that target specific communication deficits was considered important by participants. One participant raised lexical retrieval practice and a number of others spoke of scripting as approaches they actively pursued. Whilst lexical retrieval practice has a relatively large, by PPA standards, evidence base (Cadorio et al., 2017; Croot et al., 2018; Jokel et al., 2014) it is also known that this approach is more beneficial in the earlier phases of the disease journey (Cadorio et al., 2017). Participants, specifically CPs of people at advanced disease stages, explained that SLT at later stages of the disease journey would have limited benefit, due to their loved ones’ difficulty with following the sessions. To maintain communication for longer, a CP proposed strengthening less‐used skills (e.g., writing) in addition to areas of difficulty, at early disease stages, when communication difficulties are still manageable. By strengthening less‐used skills early on, this could allow for greater choice between the various compensatory approaches or strategies as symptoms progress (Khayum et al., 2012). Systematic investigation of this recommendation could provide evidence on whether this is an acceptable and effective approach. Importantly, the idea that interventions and strategies have to be fitted to a dynamically evolving disease journey is important—changing clinical priorities and changing competencies mandate flexible strategy use.

Motivation to participate in SLT is influenced by numerous factors. Adherence to lexical retrieval practice was judged as ‘painful’ albeit necessary by a participant in this study, suggesting that the anticipated outcomes may sustain motivation even when treatment is unpleasant. Thus goal‐setting may be vital to maintaining motivation in PwPPA (Oyake et al., 2020). The Self‐Determination Theory poses that goal pursuit is sustained if the psychological needs of competence, autonomy and relatedness are fulfilled (Deci & Ryan, 2000). Involving clients in the goal setting process promotes fulfilment of these needs, making it essential in maintaining high levels of motivation (Poulsen et al., 2014; Sugavanam et al., 2013). In a UK‐wide survey, speech and language therapists working with PwPPA reported goal setting to be a key component of clinical practice (Volkmer et al., 2019). Yet, lack of motivation was found to be the primary reason for cessation of lexical retrieval training in PwPPA (Taylor‐Rubin et al., 2019). Given that the behavioural variant of frontotemporal dementia, which overlaps clinically with PPA, can affect motivation and social engagement (Marshall et al., 2018) is seems likely that this additional cognitive impairment may, as participants in the focus groups highlighted, impact motivation. Anecdotal evidence also indicates comorbidities or even absence of a spouse as contributing factors (Croot et al., 2019). Identifying the contributing factors to declining motivation in PPA seems critical to better understand schedule and dosage of SLT interventions over the disease journey.

CPs in this study, most often spouses, identified their own challenges in supporting their loved ones with PPA, describing it as a puzzle. Research has emphasized that the combination of behavioural and cognitive symptoms in dementia can be difficult for CPs to manage (Pinquart & Sorenson, 2003). Participants suggested education and CP training sessions could address this so they could better support their loved ones with PPA. There are two research studies that have described education of CPs as part of a holistic SLT intervention for PwPPA (Jokel et al., 2017; Rogalski et al., 2016) and CP training interventions have demonstrated improvements in communication confidence in both the PwPPA and CPs (Volkmer, 2020c). Yet participants in the focus groups highlighted an inequity of service provision, with some CPs reporting not being involved in SLT at all. These inconsistencies in service provision were also highlighted by a UK‐wide survey of speech and language therapists (Volkmer et al., 2019). In fact, participants in this study highlight an urgent need for equitable funding for SLT services to deliver support to PwPPA across the UK.

PPA results in increased isolation, for both the person with the diagnosis and their CPs (Pozzebon et al., 2018). Indeed, all participants in the focus groups reported difficulties in participating in group conversations, describing their inability to join in family gatherings as disheartening. In addition to word finding difficulties contributing to delayed turn taking (Taylor et al., 2014), this may also be attributable to deficits in auditory processing in all PPA variants (Hardy et al., 2017; Johnson et al., 2021), where competing speech heightens auditory processing demands (Okamoto et al., 2007). Limited awareness of the disease and strategies to facilitate conversation within extended social networks was also reported by study participants. Extending CP training interventions to focus on group interactions or involving friends and family, as well as making connections with new peers or support networks, through support groups, might be useful. Whilst these approaches are not novel, and therapy groups run by NHS trusts or organizations such as Dyscover (https://www.dyscover.org.uk/), and peer support networks such as Rare Dementia Support, have been available for some time, the impact of these is difficult to measure and requires more thought in the research evidence.

### Limitations

Due to COVID‐19‐related restrictions was not possible to meet face to face at the time of the study, thus meetings were held online via a remote conferencing platform. Whilst this likely excluded some people, others commented that this negated difficult travel issues (such as mobility issues that can be significant in later PPA) which meant they would not ordinarily have been able to participate in a face‐to‐face setting. The results of this study should be interpreted whilst being mindful that this represents the views of a small sample of PwPPA or their CPs. Given that PPA is a rare disease, recruitment to research is often limited, however, the researchers sought to capture a breadth of views by ensuring representation from each PPA variant (seven nfvPPA; five svPPA; four lvPPA; one mixed PPA). Importantly many participants were CPs without communication difficulties. To guard against certain speakers (CPs) dominating over others, prompts were used to encourage equitable participation and support alternative communication modalities (e.g., non‐verbally). Though slides presented in the focus groups could have biased responses in focus groups, the research team felt the benefits of supporting comprehension for participants outweighed this risk. It is likely however, that the emotional impact of a PPA diagnosis may have been deemphasized, perhaps due to the group structure selected by the researchers. It is the authors, AV's and JW's, clinical experience that anticipatory grieving (grieving for the loss of a partner before they have passed away), guilt and a halo of other emotions impact interactions between PwPPA and their loved ones. This has also been demonstrated in the case study work published by Pozzebon et al. (2017) and highlights the need for further exploration of the emotional impact of PPA in future research.

The senior author of this study is a clinical academic speech and language therapist (AV) which may have biased the respondents. To mitigate this, the first author, who facilitated the focus groups and led the Thematic Analysis, was not a clinician and had no previous experience in working with PwPPA or in facilitating focus groups and co‐authors, AV and CH, experts in PPA, provided mentorship and training throughout the data collection and analysis phases. However, as Braun and Clarke (2006) pose, ‛data are not coded in an epistemological vacuum’ and therefore it is acknowledged that ML's, AV's and CH's experiences may have shaped the conceptualization of the specific themes and conclusions. Lastly, though participants were recruited from across England and Wales, participants do not represent a diverse group in terms of socio‐economic backgrounds, ethnicity and language spoken. Additionally, the work may be biased to those sufficiently willing, articulate, confident, logistically empowered and computer literate enough to participate. The study has taken a critical first stap in providing an invaluable picture of the needs of PwPPA and their CPs but very likely leaves vistas of the illness experience unexplored and may even have biases (see above) that will need to be redressed in future studies.

### Moving forward

This study provides a greater understanding of the experiences and needs of PwPPA and their families in relation to SLT. Interpreted alongside recent guidance from clinical academic speech and language therapists (Volkmer et al., 2022) this will enable service providers to better plan, justify funding for and deliver SLT that meet the needs of PwPPA and their families. Specifically, this work underlines the importance of a person‐centred approach, that considers the broader needs of both the PwPPA and the people around them. There is evidently much research work to be done to broaden the constituency for future work of this kind, across regions, socioeconomic strata and linguistic and cultural groups. This is the only way that SLT will really be tailored and delivered effectively to maximum benefit. This work also has much wider implications for communication difficulties in the wider spectrum of people living with dementia—a much under‐recognized issue. However, in the first instance, we urgently require guidance, in the form of care pathways to reduce the inequity in care for PwPPA and their CPs within the UK.

## CONFLICT OF INTEREST

The authors report no financial or non‐financial conflicts of interest.

## Supporting information



jlcd12818‐sup‐0001‐SuppMat.docx

## Data Availability

Videorecorded datasets generated and/or analysed during the current study are not publicly available due to ethical restrictions related to sharing of video data.
