# Supplementary material for: ‘Like going into a chocolate shop, blindfolded’: What do people with primary progressive aphasia want from speech and language therapy?
Source: Int J Lang Commun Disord. 2022 Nov 30;58(3):737–55. doi: 10.1111/1460-6984.12818 (PMC10947572; doi:10.1111/1460-6984.12818)

## **Appendix 1. Focus group topic guide.**

FG-1

12.04.21, 2.00pm

duration: 60-90 minutes

**ROLES**

Facilitator: name1: lead and facilitate the discussion, sustain conversation, prompt, clarify

Co-facilitator: name2: facilitate the discussion, prompt, when necessary

**Platform:** GoToMeeting

**2.00 pm welcome, introductions**

M: “Welcome, everyone. My name is name1.

A: [introduction]

M: “I hope everyone can see and hear us okay. Please let me know if you are experiencing any technical difficulties.

**[share slides]**

M: [SLIDE 1] “Before we start I would like to share with everyone a timetable of our session today. We will start with some introductions, and then move on to our discussion. We will have a five-minute break around 10:35”.

I would like to share a bit more about who we are and our project”.

[SLIDE 2] “I’m name1, a Master’s student. I work on this project with name2 and name3, who run the project”.

[SLIDE 3] “We all work with UCL and the Rare Dementia Support. This project is part of the RDS Impact Study, which is a five-year study for people with rare forms of dementia.”

[SLIDE 4] “Before we start, I would like to remind you that this meeting will be recorded. If you don’t wish to be recorded, you can leave the meeting now”.

[SLIDE 5] “The recording will be used for research and will be stored safely”.

[SLIDE 6] “Direct quotes may be used in published research, but your identity will be protected. If it’s okay with everyone, I’m going to start recording the meeting now”.

**[start recording]**

[SLIDE 7] “Today, we are interested in hearing your suggestions about what speech and language therapies you believe would be most useful to you. You can think about your current experience with speech and language therapy, what was useful and what you think can help you in the future”.

**[stop screen sharing]**

“I would like to thank you all for agreeing to participate in this project. Before we start our discussion, I would like to invite you to introduce yourselves to the group, by telling us your first name”.

“It is lovely to meet everyone. I would like to assure you that this is a safe space, where everyone is free to share and be honest. Your opinions will be respected by everyone in the group”.

**[share slides]**

[SLIDE 7] “As I said, today we want to hear your opinions on what speech and language therapies you think would be most useful to you. To help you think about this, we have some ideas, but you are free to discuss your own ideas”.

[SLIDE 8] “Some people might have difficulties following what someone is saying. Maybe these people would like practising listening exercises. For example, listening to very short stories or sentences, and then discussing those with a partner. *I’m going to start with [people with PPA] first, and then move on to partners*. What do you think of this therapy?”

**[stop sharing screen, allow participants to engage in conversation for approximately 5-10 minutes]**

“Thank you for sharing your ideas”.

[SLIDE 9] “When talking with a partner, some people might have difficulty responding appropriately. Maybe these people would like practising words and sentences. What do you think of this idea? [People with PPA] do you have anything to add?”

**[stop sharing screen, allow participants to engage in conversation for approximately 5-10 minutes]**

“Thank you for your ideas everyone. At this point I think we can have a five-minute break, and come back at **2:40**”. **[stop recording] show slide 10**

**[start recording]**

“Welcome back! If everyone’s ready we can continue”.

[SLIDE 11] “Other people might have difficulty following complex sentences when talking with someone. These people might prefer practising writing exercises. [People with PPA] what do you think of this idea?”

**[stop sharing screen, allow participants to engage in conversation for approximately 5-10 minutes]**

“Thank you for these ideas everyone, they were very helpful”.

[SLIDE 12] “Some people may also have difficulties with words that sound very similar. Maybe practising these words can help? What do you think?”

**[stop sharing screen, allow participants to engage in conversation for approximately 5-10 minutes]**

“Thank you for sharing everyone”

[SLIDE 13] “Some people may prefer help with specific daily life activities. For example, ordering coffee, talking about their day to a friend or family member, or reading the newspaper. What would you like to practise in speech therapy to help you in your daily life?

**[stop screen sharing]**

**3.10pm: Thank participants**

“I would like to thank you for your time today. You shared some very interesting ideas with us. We are happy to answer any questions or comments, so please get in touch.”

**Prompts to sustain conversation or for participants who may not engage spontaneously**

- [Person 1] do you have anything else to add?
- Thank you for sharing [partner 1]. [Person 2] do have anything else to add?
- [Person 1] what do you think about [intervention]?
- Thank you for this idea [person 1]. [Person 2] would you like [intervention]?
- Thank you [partner 1]. [Person 2], what do you think?
- Thank you for your ideas, partners. [People with PPA] do you think [intervention] would be helpful to you?
- [Person1]?
- [summarise what has been said]
- What about therapy for you as a partner?
- What would you like to have been offered in the past?


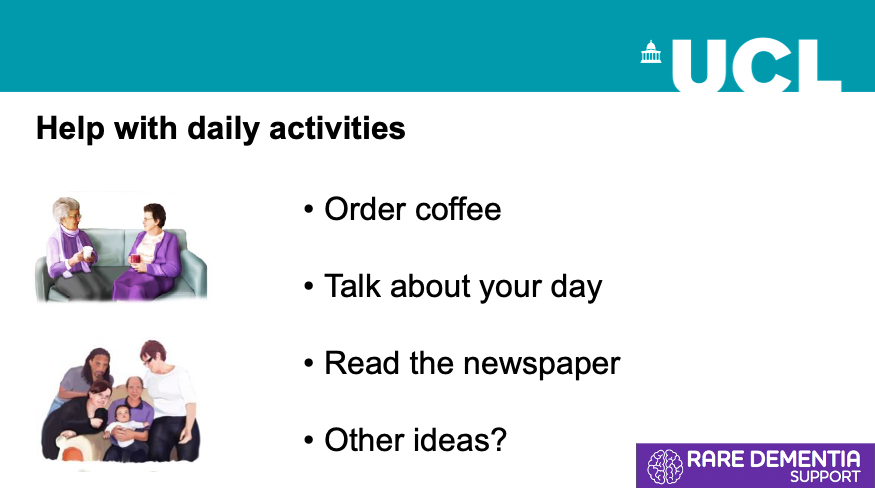
Example slides:


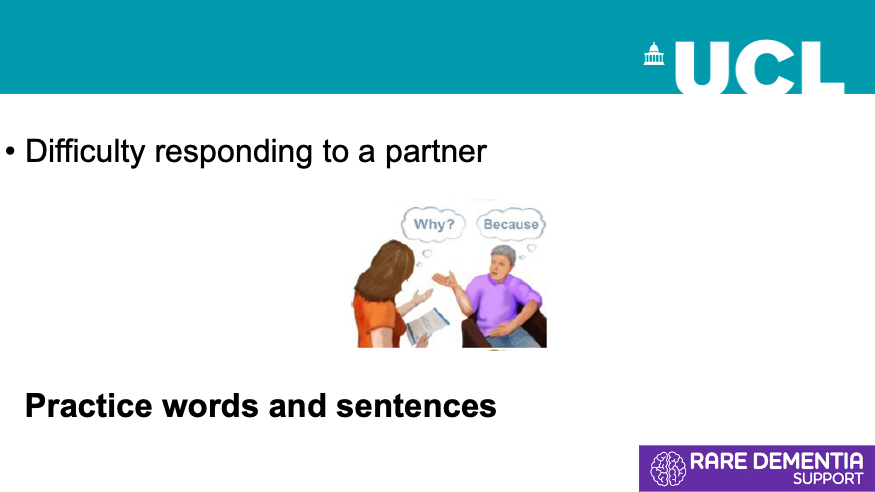

Supplement: Supplementary file 1 — jlcd12818‐sup‐0001‐SuppMat.docx [file JLCD-58-737-s001.docx]
